# Genome-Wide Identification and Characterization of the OPR Gene Family in Wheat (*Triticum aestivum* L.)

**DOI:** 10.3390/ijms20081914

**Published:** 2019-04-18

**Authors:** Yifei Mou, Yuanyuan Liu, Shujun Tian, Qiping Guo, Chengshe Wang, Shanshan Wen

**Affiliations:** College of Agronomy, Northwest A&F University, Yangling 712100, Shaanxi, China; mouyifei@nwsuaf.edu.cn (Y.M.); yuanyuan_L1994@163.com (Y.L.); tianshushu666@163.com (S.T.); nwafungqp@163.com (Q.G.)

**Keywords:** wheat, OPR, gene family, expression pattern, stress response

## Abstract

The 12-oxo-phytodienoic acid reductases (OPRs), which belong to the old yellow enzyme (OYE) family, are flavin mononucleotide (FMN)-dependent oxidoreductases with critical functions in plants. Despite the clear characteristics of growth and development, as well as the defense responses in *Arabidopsis*, tomato, rice, and maize, the potential roles of OPRs in wheat are not fully understood. Here, forty-eight putative *OPR* genes were found and classified into five subfamilies, with 6 in sub. I, 4 in sub. II, 33 in sub. III, 3 in sub. IV, and 2 in sub. V. Similar gene structures and conserved protein motifs of TaOPRs in wheat were identified in the same subfamilies. An analysis of cis-acting elements in promoters revealed that the functions of OPRs in wheat were mostly related to growth, development, hormones, biotic, and abiotic stresses. A total of 14 wheat *OPR* genes were identified as tandem duplicated genes, while 37 *OPR* genes were segmentally duplicated genes. The expression patterns of TaOPRs were tissue- and stress-specific, and the expression of TaOPRs could be regulated or induced by phytohormones and various stresses. Therefore, there were multiple wheat *OPR* genes, classified into five subfamilies, with functional diversification and specific expression patterns, and to our knowledge, this was the first study to systematically investigate the wheat *OPR* gene family. The findings not only provide a scientific foundation for the comprehensive understanding of the wheat *OPR* gene family, but could also be helpful for screening more candidate genes and breeding new varieties of wheat, with a high yield and stress resistance.

## 1. Introduction

Jasmonates (JAs) are a kind of lipid-derived signaling molecules in plants and function in response to various stresses, as well as plant growth and development [1,2]. Jasmonic acid and related derivatives act as regulators in defense against biotic and abiotic stresses, such as the attack of the hessian fly, aphid, pathogen, salt, low and high temperatures, and wounding [3,4]. Besides, they also play a vital role in tendril coiling, fruit ripening, pollen maturation, root growth, and seed germination [5,6]. The conversion from cis-12-oxo-phytodienoic acid (OPDA) to 12-oxo-phytoenoic acid (OPC-8:0), catalyzed by OPRs, is a key process in the JA biosynthesis pathway [1].

*OPR* genes belong to the OYE family and are classified as FMN-dependent oxidoreductases, with multiple subfamilies in plants [7]. The Oxidored_FMN (ID: PF00724) was the only domain in the typical OPR proteins via the Pfam analysis. According to the phylogenetic analysis of six major green plants (green algae, mosses, lycophytes, gymnosperms, monocots, and dicots), the *OPR* genes were classified into seven conserved subfamilies. Furthermore, two subfamilies in dicots (sub. I and II) and five subfamilies (sub. IV) in monocots were discovered [8]. In previous studies, based on the substrate specificity, the OPRs in *Arabidopsis thaliana* were divided into two groups, group I (OPRI) and group II (OPRII) [9]. The *AtOPR1* and *AtOPR2* (OPRI) preferentially catalyze (9R,13R)-12-oxophytodienoic acid (9R,13R-OPDA), which is not involved in the JA biosynthetic process. The *AtOPR3* belonging to OPRII can convert the 9S,13S-OPDA to oxo-2(2’(Z)-pentenyl)-cyclopentane-1-octanoic acid (OPC-8:0), and OPC-8:0 is the precursor of JA biosynthesis [10,11]. Then, the classification of OPR subfamilies in monocot rice, especially for the sub. III, IV, and V, was comprehensively conducted by Li et al. (2011) [12]. The difference of (three-dimensional) 3D structure of rice OPR proteins, typically two middle variable regions (MVR i and ii), decided the substrate specificity and catalytic activity of different subfamily OPR proteins [12]. In specific, similar 3D structures, except in MVR ii, were found in Sub. I, II, and V, and rice OPR proteins in Sub. I and II showed strong or moderate catalytic activity with substrates, while the sub. V presented weak catalytic activity. Besides, the rice OPR proteins in Sub. III and IV had similar 3D structures, but could be distinguished by the difference of MVR i [12].

To date, the biochemical and physiological functions of OPRs have been reported in dicots and monocots. In dicot *Arabidopsis*, the OPRs can be activated by wounding, pathogens, cadmium ion, and hormone signaling molecules, such as JA, abscisic acid (ABA), salicylic acid (SA), and ethylene (ET) [13,14]. The expression profiles of genes in the subfamilies OPRI and OPRII in *Arabidopsis* and *Nicotiana tabacum L.* plants presented tissue-specific patterns [15]. *AtOPR1* was expressed in young seeds, while *AtOPR2* was detected in pollen, and both *AtOPR1* and *AtOPR2* were found in roots [15]. In addition, *AtOPR3* was expressed in various tissues of the *Arabidopsis* [16]. A peroxisomal signal peptide was found in *AtOPR3* (OPRII). Thus, *AtOPR3* can be localized in the peroxisome. However, this specific peroxisomal target sequence was not discovered in OPRI [16,17]. The capacity of transporting into peroxisomes is important for the subcellular location of *OPR* and the subsequent signaling pathway [18]. An individual *OPR* may exhibit multi-functions, owing to the substrate specificity, subcellular location, and differential expression in different tissues and specific stresses [19]. For instance, in maize, the *ZmOPR1* and *ZmOPR2* defenses against pathogens were induced by SA and chitooligosaccharides, not by wounding, while *ZmOPR7* and *ZmOPR8* were induced by wounding, as well as crosstalk with signaling molecules, such as JA, ABA, and SA [20]. It was noteworthy that two wheat *OPR* genes belonging to sub. I and sub. II have been reported, and they could enhance wheat tolerance to abiotic stresses (salt, wounding, drought, et al.) through the ABA-dependent pathway or the JA biosynthesis pathway [21,22].

The functions of OPRI and OPRII have been well characterized in dicot plants [15,23,24,25]. However, the OPRs can be divided into five subfamilies in monocots, and the functions of OPRI and OPRII have not been fully understood, let alone the other subfamilies III-V [8]. Thus, a genome-wide analysis of OPRs is essential for understanding the function of the *OPR* gene family in wheat. In this study, the wheat *OPR* gene family was identified via a genome-wide search. Then, the putative 48 wheat *OPR* genes were systematically analyzed, including the gene phylogenetic relationship, gene structure, protein conserved domain, chromosome localization, cis-acting regulatory elements, and expression profiles, to indicate the evolutionary and functional features of these genes. In addition, the relative expression of five *OPR* genes from five different subfamilies (2 tissues and eight stresses for each gene) were determined using qRT-PCR to better comprehend the *OPR* functions in growth and development, as well as in stress responses. This work could contribute to the selection of more candidate genes for further functional study of *OPR* genes in order to improve resistance against various stresses.

## 2. Results

### 2.1. Genome-Wide Identification of the OPR Gene Family in Wheat

A total of 55 TaOPRs were identified by local BLASTP using HMM profiles, and at last, 48 putative TaOPRs were confirmed by detecting the *Oxidored_FMN* conserved domain via Pfam and the NCBI-CD database. From the TAIR database, 3 *Arabidopsis* OPRs were obtained, and 8 maize OPRs and 13 rice OPRs were explored and compared against NCBI by the key word, 12-oxo-phytodienoic acid reductase. *Arabidopsis*, maize, and rice *OPR* gene IDs were available in Table S2. The 72 OPR-like proteins were used for further phylogenetic analysis (Table 1).

The Ensembl wheat gene ID, subfamily gene, amino acid length, PI, MW, subcellular location and location were presented in Table S1. The lengths of TaOPR proteins ranged from 204 to 399 amino acids, the PI ranged from 4.84 to 8.23, and the molecular weight ranged from 23.64 to 44.14 kDa. The predicted subcellular localization analysis showed that only 3 TaOPRs were localized in peroxisome (*TaOPRII-B1*, *TaOPRIII-D13*, and *TaOPRIII-B9*). In sub. I, the *TaOPRI-D2* and *TaOPRI-A3* were predicted to be localized in mitochondrial, and the other four TaOPRI proteins were localized in the chloroplast. In sub. III, IV, and V, a total of 17 OPRs were predicted to be localized in mitochondria, and 19 OPRs were localized in cytoplasms (Table S1). Additionally, the prediction of subcellular localization of *OPR* genes should be further validated in vivo.

### 2.2. Classification and Phylogenetic Analysis of TaOPRs

**Figure 1:**
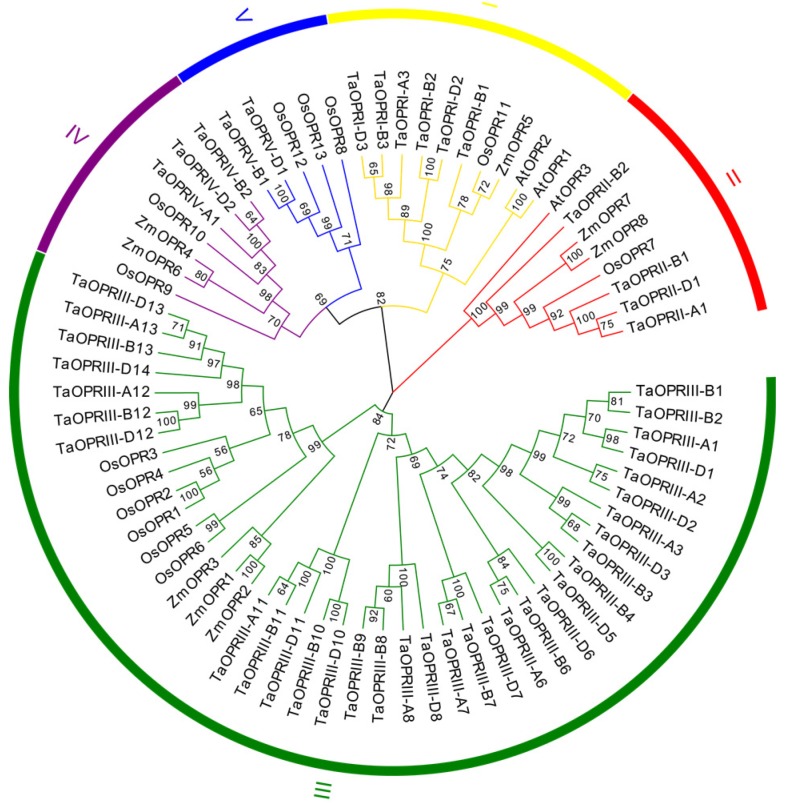
Phylogenetic tree of the wheat *OPR* family. The maximum-likelihood (ML) phylogenetic tree was constructed based on the amino acid sequence alignments of wheat (48), *Arabidopsis* (3), *Oryza sativa* (13), and *Zea mays* (8), using the MEGA7.0 software, with 1000 replicates. *OPR* genes in wheat are classified into five subfamilies, and the names of each subfamily are shown in different colors outside of the circle.

To further investigate the phylogenetic relationships and predict the classification of the wheat *OPR* genes, a maximum-likelihood phylogenetic tree was constructed using 72 conserved OPR proteins blocks of *Arabidopsis*, rice, maize, and wheat by MEGA 7.0. The conserved protein blocks were selected using Gblocks Server. The best-fit model to construct the tree was WAG+I+G (gramma shape = 1.361 and proportion of invariable sites = 0.018). On the basis of the classification of other plants and the amino acid structure of OPRs [8], the TaOPRs were classified into five subfamilies (sub. I-V) and named OPRI-V (Figure 1). Interestingly, there were 33 proteins in sub. III, and it was the largest subfamily of OPRs, while the number of proteins in the other four subfamilies was 6 in sub. I, 4 in sub. II, 3 in sub. IV, and 2 in sub. V.

In addition, TaOPRI-B1, TaOPRI-B2, TaOPRI-D2, TaOPRI-A3, TaOPRI-B3, and TaOPRI-D3 were closely related to AtOPR1, AtOPR2, ZmOPR5, and OsOPR11 and were clustered in sub. I, and TaOPRII-A1, TaOPRII-B1, TaOPRII-D1, and TaOPRII-B2 were tightly linked to AtOPR3, ZmOPR7, ZmOPR8, and OsOPR7 and were clustered in sub. II. Wheat sub. III was clustered with ZmOPR1-3, and OsOPR1-6. Wheat sub. IV was tightly related to ZmOPR4, ZmOPR6, OsOPR9, and OsOPR10. Wheat sub. V was clustered with OsOPR8, OsOPR12, and OsOPR13. In previous studies, the physiological biochemical character of the *OPR* gene family in the *Arabidopsis* of sub. I and sub. II was different [15,16,26]. However, the physiological and biochemical functions in sub. III, IV, and V of monocots remained unclear. In addition, differences of evolutionary rates resulted in various functions of *OPR* genes, and the *OPR* subfamily, generated later, evolved some new subgroup-specific functions after divergence [8]. In consequence, the functions of the *OPR* family genes were diverse in different subfamilies. While there were conserved domains in OPRs, the gene functions might be significantly different. This was consistent with the OPRs in maize [20] and rice [12].

### 2.3. Gene Structure and Protein-Conserved Domains Analysis of TaOPRs

**Figure 2:**
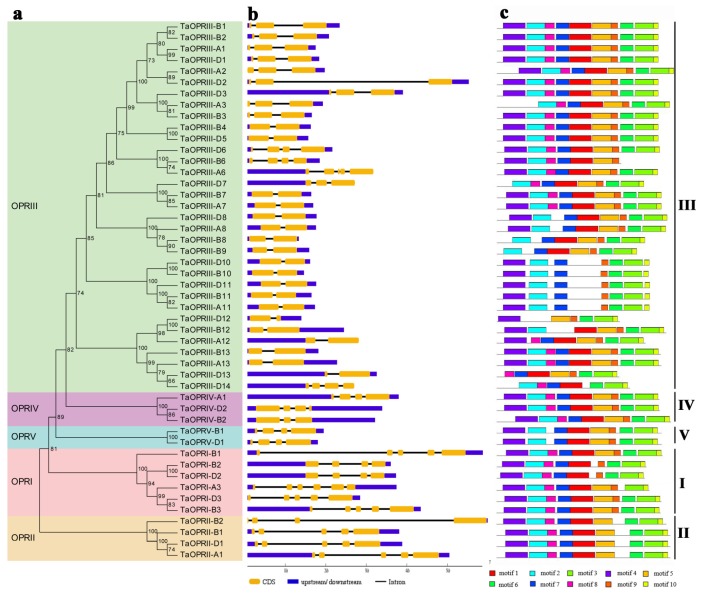
Phylogenetic relationship, gene structure, and conserved motif analysis of *TaOPR* genes. (**a**) Phylogenetic tree of 48 wheat OPR proteins. The maximum-likelihood phylogenetic tree was constructed using MEGA7.0, with 1000 replicates. (**b**) Exon-intron structures of *TaOPR* genes. Orange boxes represent exons, black lines represent introns, and the upstream/downstream regions of *TaOPR* genes are represented by blue boxes. (**c**) Conserved motifs of TaOPR proteins. Ten conserved motifs are shown in different colored boxes, and the details of the motifs are provided in Table S3.

To further estimate the gene structure and protein-conserved domains of wheat *OPR* genes, the conserved protein blocks of the 48 TaOPRs were aligned using ClustalW, and the maximum-likelihood method was used to construct a phylogenetic tree (Figure 2a). The OPRs in wheat were classified into five subfamilies, which were clustered in a similar way to those shown in Figure 1. The protein-conserved domains of 48 wheat *OPR* genes were identified by searching and comparing them against MEME Suite databases. At most, 10 conserved motifs were found in each OPR. The lengths of these motifs varied from 11 to 50 amino acids, and the details of the 10 conserved motifs were presented in the Table S3. As exhibited in Figure 2c, OPRs were highly conserved and 23 OPRs had 10 conserved motifs, including 3 in sub. I, 3 in sub. IV, and 17 in sub. III. In addition, sub. II lacked motif 8, which completely corresponded to the β6 barrel of the protein secondary structures, and sub. V lacked motif 9, which corresponded to the βC barrel. In sub. III, most of the OPR proteins had 10 conserved motifs, but some of them lacked 1 to 4 motifs.

The exon/intron structures of *OPR* genes were further analyzed and presented by the GSDS (Gene Structure Display Server)(Figure 2b). We found that the exon/intron structures in the *TaOPR* genes varied among diverse subfamilies but were relatively conserved within the same subfamily. As shown in Figure 2b, the intron numbers and lengths in different subfamilies were notably different. Most of the *OPR* genes in subfamilies I, II, IV, and V contained 3–5 introns. In subfamily III, only *TaOPRIII-D14*, *TaOPRIII-D6*, *TaOPRIII-A6*, and *TaOPRIII-B6* had three introns, and the other 29 contained 1–2 introns.

### 2.4. Cis-Acting Elements in the Promoters of TaOPRs

**Figure 3:**
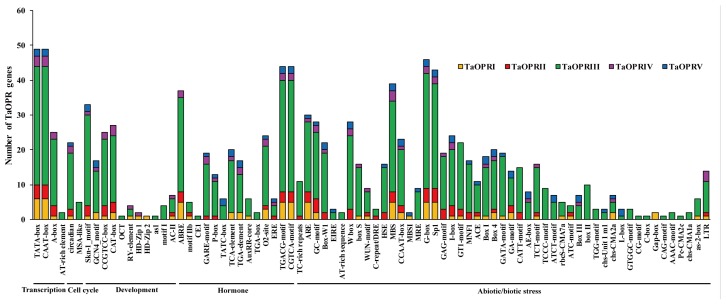
Number of various cis-acting regulatory elements of *TaOPR* genes. The cis-acting regulatory elements were identified from PlantCARE online by an analysis at 1.5 kb upstream of the transcription start site of *TaOPR* genes. The graph was generated using cis-acting element names and functions of *TaOPR* genes, and the number of elements in five different subfamilies are shown in different colors.

Cis-acting elements in the promoter are crucial regions of the binding site of the transcription factors for initiating transcription, which plays a vital role in regulating gene expression. To further explore the possible biological functions of OPRs, the 1.5 kb upstream promoter regions of all OPRs were used to predict the cis-acting regulatory elements via the online database, PlantCARE. Accordingly, various cis-acting regulatory elements, predicted to be related to transcription, cell cycle, development, hormones, and stresses, were discovered in the promoter regions of wheat *OPR* genes (Figure 3). Some elements were related to root-specific leaf morphology, seed-specific, meristem-specific, and endosperm expression, and the number of elements related to seed-specific leaf morphology and root-specific expression was 4, 1, and 5, respectively. Besides, many cis-acting elements related to hormone signaling pathways were found, such as methyl jasmonate (MeJA), ABA, SA, gibberellins (GA), auxin (IAA), and ET. A total of 44 OPRs related to MeJA-responsive elements, containing TGACG-motif and CGTCA-motif, were discovered, and the number of ABA-responsive elements (ABRE) was 37, indicating that most of the OPRs might participate in JA- and ABA-mediated signaling pathways. In addition, a few elements were predicted to be involved in various abiotic stresses, such as wounding, drought, salt, heat, cold, light, and fungus. In particular, all of the *OPR* genes contained light-responsive elements.

### 2.5. Chromosomal Location and Gene Duplication of TaOPRs

According to the available wheat genome annotation information, a total of 48 wheat *OPR* genes were mapped onto 15 of the 21 wheat chromosomes. The wheat gene ID of EnsemblPlants confirmed the chromosome localization (Table S1). There were 12, 19, and 17 *OPR* genes located on the A, B, and D sub-genome, respectively. Six *OPR* genes were distributed in chromosomes 1B, 1D, and 7D, 5 *OPR* genes in chromosomes 1A and 7B, and 3 *OPR* genes in chromosomes 2B and 2D. Besides, two *OPR* genes in chromosomes 2A, 4B, and 6B, as well as only one gene in chromosomes 4D, 5A, 5B, and 6D, were identified. In contrast, no *OPR* gene was found in chromosomes 3A, 3B, 3D, 4A, 5D, and 6A. Hence, the distribution of *OPR* genes was not homogeneous and random in wheat chromosomes, indicating that gene duplication events might occur in wheat chromosomes 1 (A, B, D) and 7 (A, B, D) during evolution.

**Figure 4:**
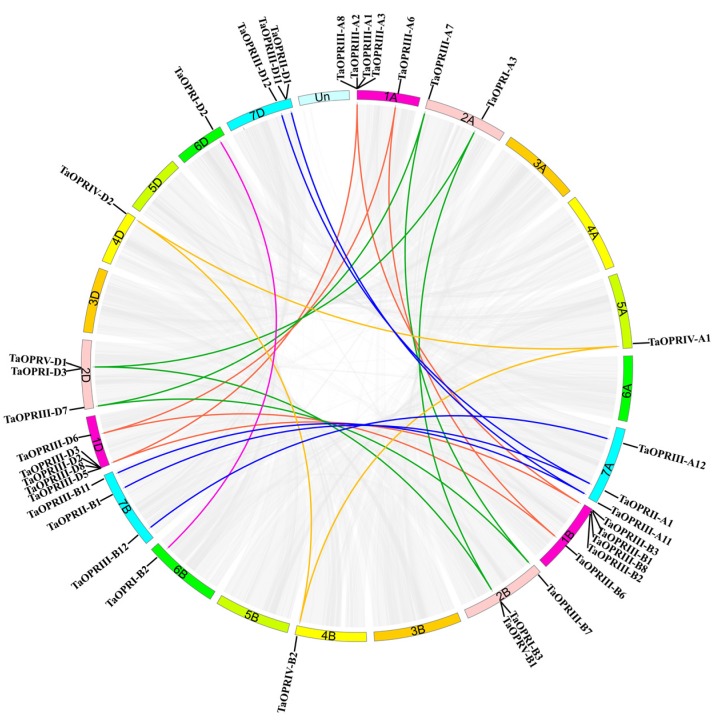
Genomic distribution of *TaOPR* genes and gene homology analysis in wheat. Gray lines are all synteny blocks in the wheat genome, and the different color lines indicate duplicated *OPR* gene pairs on different chromosome. 1A, 1B, 1D indicate chromosome 1A, chromosome 1B and chromosome 1D, respectively.

The tandem and segmental duplications of genes were widespread in plant genomes. Thus, we analyzed the duplication events of wheat *OPR* genes. A total of 14 wheat *OPR* genes were identified as tandem duplicated genes and located in chromosomes 1 (A, B, D) and 7 (A, B, D). Additionally, 4 groups of two tandem duplicated genes were located in chromosomes 1D, 7A, 7B, and 7D, and 2 groups of three tandem duplicated genes were located in chromosomes 1A and 1B (Table S4). Furthermore, 32 gene pairs (37 *OPR* genes) were segmentally duplicated genes, including 30 pairs (36 *OPR* genes) of homoeologous genes, and were distributed in different chromosomes (Figure 4, Table S5). In this study, there were a total of 10 homoeologous gene groups with a copy on each A, B, and D chromosome, and 3 gene groups with a copy on only 2 of the 3 chromosomes. There were still 7 OPRs that were neither tandem nor segmentally duplicated genes. The above results indicated that the tandem and segmental duplication events were essential for the expansion of the *OPR* gene family, and the segmental duplication seemed to play the predominant role.

### 2.6. Tissue-Specific Expression Patterns of TaOPRs

**Figure 5:**
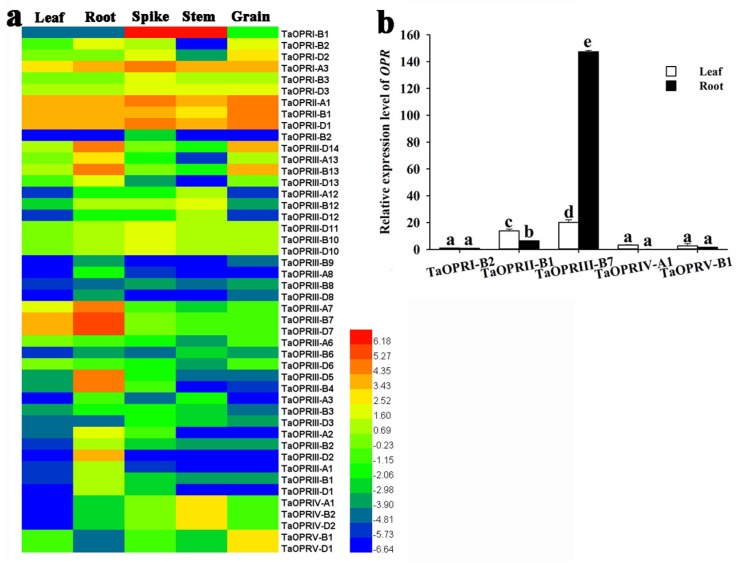
(**a**) Expression profiles of TaOPRs in five different tissues and organs (leaf, root, spike, stem, and grain). The heatmap was constructed using the Heml software, and the FPKM (fragments per kilobase of transcript per million fragments) values of *TaOPR* genes were transformed by log2. The red and blue colors represent the higher and lower relative abundance of the transcript, respectively. (**b**) Analysis of expression profiles of five *TaOPR* genes in leaf and root by qRT-PCR. Data were presented as mean ± SD (*n* = 3), and the values differed significantly when *p* < 0.05. Varied letters within a figure meant a significant difference.

Based on the available RNA-seq databases, the temporal and spatial expression patterns of 46 wheat *OPR* genes in 5 different tissues were visualized using the Heml software (Figure 5a). The expression levels of TaOPRs varied significantly in different tissues. For ease of description, the homoeologous genes on different chromosomes (chr A, B, and D) were denoted as *TaOPR*-subfamily-gene. For example, the *TaOPRI-A3*, *TaOPRI-B3*, and *TaOPRI-D3* were all named as *TaOPRI-3*. All the TaOPRs, except *TaOPRII-2*, were expressed in at least 2 tissues. The sub. II family member, *TaOPRII-1*, was widely expressed in all of the 5 tissues. The expression level of *TaOPRII-2* was very low in the root, and no expression was detected in the leaf, stem, and grain. Three genes, including *TaOPRIII-2*, *TaOPRIII-4*, and *TaOPRIII-5*, were mainly expressed in the root. The *TaOPRIII-7* was significantly highly expressed in leaf and root, and the *TaOPRIII-14* showed preferential expression in root and grain. In addition, the *TaOPRI-1* was extremely highly expressed in the spike and stem. As shown in Figure 5a and Table S7, most of the homoeologous genes exhibited similar expression patterns, while some of them presented diverse expression patterns. For instance, *TaOPRIII-B13* was predominantly expressed in root and grain, while its homoeologous genes *TaOPRIII-A13* and *TaOPRIII-D13* were only expressed in root. It was noteworthy that, in the sub. III family, a total of 4 OPR genes presented very low expression abundances in five tissues. 

Two tissues (leaf and root) of three-leaf-stage seedlings were used to determine the tissue-specific expression patterns by qRT-PCR (Figure 5b). Based on the result of qRT-PCR, the expression of *TaOPRII-B1* and *TaOPRIII-B7* were extremely high in these two tissues, and the expression of *TaOPRIII-B7* was the highest in root, while *TaOPRI-B2*, *TaOPRIV-A1*, and *TaOPRV-B1* showed significantly lower expression abundances. These results indicated that the expression patterns of TaOPRs were tissue-specific.

### 2.7. Expression Patterns of TaOPRs under Abiotic Stresses

**Figure 6:**
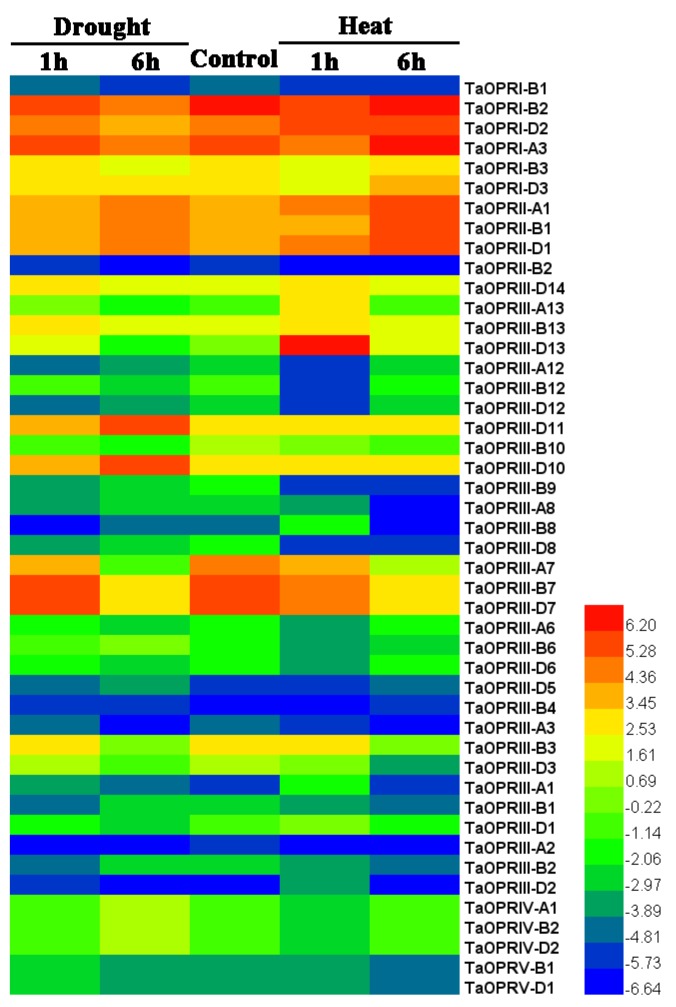
The expression profiles of 46 *TaOPR* genes under drought and heat stress treatments. FPKM (fragments per kilobase of transcript per million fragments)values of *TaOPR* genes were transformed by log2 to create the heat map using the Heml software. The red and blue colors represent the higher and lower relative abundance of each *OPR* gene, respectively.

In order to understand the expression profiles of TaOPRs under abiotic stresses, we used the available RNA-seq data to study the expression of wheat OPRs in response to drought and heat stresses. According to the log2 FPKM values, the *OPR* genes showed differential expression patterns under drought and heat stresses (Figure 6). Four TaOPRs, including *TaOPRI-2*, *TaOPRI-3*, *TaOPRII-1*, and *TaOPRIII-7*, were highly expressed in all treatments. Some genes showed opposite expression patterns under the same stress. For example, the expression of *TaOPRII-1* and *TaOPRIII-11* were up-regulated after 1 h and 6h of drought stress, while the expression of *TaOPRI-2* and *TaOPRIII-7* were down-regulated after 6 h of drought stress. The expression level of *TaOPRII-1* was increased, and the expression level of *TaOPRIII-7* was decreased after heat stress. Besides, there were some *OPR* genes presenting low expression abundances in both the control and stress treatments. It is worth mentioning that most of the homoeologous genes showed similar expression patterns in response to stresses, while there were still a few genes presenting converse expression patterns, including *TaOPRIII-10* and *TaOPRIII-6* under drought stress. Particularly, the expression level of *TaOPRIII-D13* was increased sharply after 1 h of heat stress.

### 2.8. Expression of TaOPRs under Different Stresses via qRT-PCR

**Figure 7:**
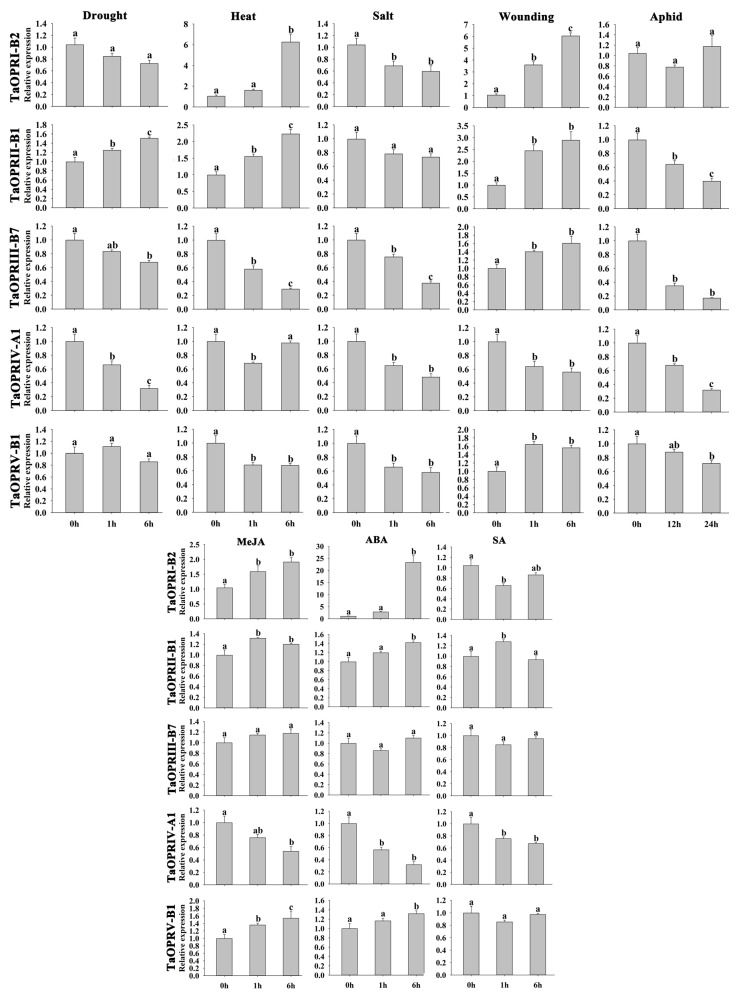
Analysis of the relative expression level of TaOPRs by qRT-PCR. Expression profiles of five *TaOPR* genes, *TaOPRI-B2*, *TaOPRII-B1*, *TaOPRIII-B7*, *TaOPRIV-A1*, and *TaOPRV-B1*, under drought, heat, salt, wound, MeJA, ABA, SA, and aphid stresses. Data were presented as mean ± SD (*n* = 3), and the values differed significantly when *p* < 0.05. Varied letters within a figure meant a significant difference.

To further ascertain the biological functions of *OPR* genes in different subfamilies, we determined the expression profiles of 5 genes using three-leaf-stage leaves in the different subfamily (I–V), under 8 stress treatments, including abiotic stresses (drought, heat, salt, wounding), hormonal treatments (MeJA, ABA, SA), and biotic stress (aphid). The relative expression of *TaOPR* genes in five subfamilies (I, II, III, IV, and V) showed various expression profiles (Figure 7). Overall, these five TaOPRs could be induced by almost all the treatments. For instance, *TaOPRIV-A1* was decreased significantly by these eight treatments at two time points, except after 6h under heat stress. *TaOPRIII-B7* was induced by all abiotic and biotic stresses, except three hormonal treatments. The expression of some genes, such as *TaOPRI-B2* under drought and aphid, *TaOPRII-B1* under salt, and *TaOPRV-B1* under drought and SA, showed no significant change. Interestingly, several genes exhibited opposite expression patterns under different treatments. For example, *TaOPRI-B2* was highly up-regulated by heat, wounding, MeJA, and ABA treatments, while it was significantly down-regulated under salt and SA stresses. The expression of *TaOPRIII-B7* decreased obviously under drought, heat, salt, and aphid stresses, but notably increased in response to wounding. In addition, genes within different subfamilies showed similar expression patterns under the same treatments. For instance, four genes (*TaOPRI-B2*, *TaOPRIII-B7*, *TaOPRIV-A1,* and *TaOPRV-B1*) were down-regulated under salt stress, and the *TaOPRII-B1*, *TaOPRIII-B7*, *TaOPRIV-A1*, and *TaOPRV-B1* were negatively regulated by aphid treatment. Nevertheless, some genes in different subfamilies exhibited opposite expression patterns under drought, heat, wounding, MeJA, ABA, and SA treatments. 

## 3. Discussion

OPRs are multigene families and can be identified in many plants [8]. While previous studies have focused on the structure, evolution, and functional characterization of OPRs in *Arabidopsis* [15], tomato [18], maize [20], rice [12], and *Camellia sinensis* [27], they have not been studied in wheat. Eight *OPR* genes were identified from maize, and a series of analyses revealed that various *OPR* genes were located in the cytoplasm or peroxisome, performing different functions, and were differentially regulated in response to stress-associated hormones, wounding, or pathogen infections [20]. In rice, 13 *OPR* genes were identified, and the expression profiles obviously showed tissue-specific and stress-specific elements, such as hormones, wounding, and other abiotic or biotic stresses [12]. In wheat, there has been no comprehensive study focusing on the *OPR* genes, but the previous work of *TaOPR1* and *TaOPR2* could provide an insight on functions of OPRs [21,22].

In this study, a total of 48 TaOPRs were identified from wheat and characterized. The 48 TaOPRs were clustered into five subfamilies (named as sub. I-V), which is consistent with Li et al. (2009). According to the phylogenetic and gene structure analysis, most of the *OPR* genes within a subfamily showed a similar exon/intron structure, indicating that the evolution might not only affect the gene function, but also the gene structure [28,29]. Subfamily III was the youngest subfamily, containing the fewest introns. Thus, it could be speculated that the intron loss events occurred during the structural evolution of the *OPR* gene family [8]. The diversity of protein-conserved motifs was found in wheat OPRs. As shown in Figure 2c, the TaOPRs in sub. II and sub. V lacked motif 8 and 9, respectively, and the absent motifs were crucial for the secondary structure of proteins [27]. Analogously, the motif loss events were also identified in sub. III, and the number and position were diverse as well. Taking the results of expression patterns under drought stress together, we discovered five genes in sub. III (*TaOPPRIII-B10*, *TaOPPRIII-D10*, *TaOPPRIII-A11*, *TaOPPRIII-B11*, and *TaOPPRIII-D11*) which lacked motif 1, 5, and 8, and they presented converse expression patterns with other genes possessing the whole motifs. The similar trends were found for most of the other genes that lacked motifs. Therefore, the diversity of protein structure probably contributed to diverse expression patterns. As a result of the selection pressures during genome evolution in wheat, the *OPR* genes within the same subfamily gradually formed various gene structures or protein motifs in order to respond to various conditions [30].

Comparisons of wheat OPR proteins with other plant species (rice, maize, and *Arabidopsis*) were conducted to investigate the evolution of the OPR family. We found 13 and 8 proteins in monocot rice and maize, respectively, and 3 OPR proteins in dicot *Arabidopsis.* Thus, the number of OPR proteins in wheat (48 TaOPRs) was much higher than that in rice, maize. and *Arabidopsis*. The reason might be attributed to the allohexaploid genome and complex evolution in wheat [31,32]. Furthermore, the wheat experienced 2 whole genome duplication events from donors of the A, B, and D genomes [33].

The gene duplication events were usually derived from the polyploidization or tandem and segmental duplication, which contributed to the expansion of gene families and genome evolution [30,31]. We found 14 tandem duplication and 37 segmental duplication *OPR* genes, and they were distributed in all subfamilies. It seemed to be true that in comparison with the tandem duplications, more segmental duplications occurred in the *OPR* gene family. Additionally, all the tandem duplicated OPRs and 24 of the 37 segmentally duplicated OPRs belonged to subfamily III. Interestingly, there were 36 homoeologous genes in 37 segmentally duplicated OPRs, and not every OPR had three homoeologous genes on the homologous chromosomes A, B, and D. There were 3 pairs of TaOPRs with two homoeologous genes in wheat. Supporting our findings, it had been reported that some homologous genes could be lost during the polyploidization of the genome [34]. Hence, it was highly possible that the gene duplication led to the expansion of the *OPR* gene family, and these new genes contributed to the formation of new biological functions [35].

Based on the predictive analysis of cis-acting regulatory elements, the OPRs might involve regulating various biological processes. In our study, the wheat OPRs were closely associated with the stimulation of phytohormones, including MeJA, ABA, SA, GA, IAA, and ET, as well as responses to various stresses (wound, drought, salt, heat, cold, light, and fungus). Thus, it could be speculated that the wheat *OPR* genes participated in specific signaling pathways to regulate the growth, development, and defensive responses. For instance, *TaOPR1* (sub. I) could enhance salinity tolerance via an ABA-dependent signaling pathway, and *TaOPR2* (sub. II) was involved in the JA biosynthesis pathway and could be induced by various stresses (wounding, drought, MeJA, and *Puccinia recondite f. sp. tritici*) [21,22]. In addition, the *TaOPR2* could rescue the male sterility phenotype of *Arabidopsis* mutant *opr3*, thereby it played a vital role in the male development of wheat. It has been reported, in several studies, that OPRs participated in the JA signaling pathway to defend against phloem-feeding insects, such as aphids and brown planthoppers [36,37]. Moreover, OPRs in *Arabidopsis* can modulate the stomatal closure to defend against drought stress by affecting the production of OPDA (cis-12-oxo-phytodienoic acid), a precursor of OPR, and this process was closely related to JA and ABA [4]. Supporting our results, the OPRs have been found to regulate a series of responses to biotic and abiotic stresses (salt, wound, ROS-scavenging, herbivore defenses, etc.) in tomato, rice, and *Arabidopsis* [18,38,39]. Therefore, the results indicated that TaOPRs might participate in the development and stress resistance through hormone-specific signaling pathways.

The expression profiles of *OPR* genes in wheat were diverse in different tissues and developmental periods, which could be inferred from the analyses of qRT-PCR and public transcriptome data. In *Arabidopsis*, the two *OPR* genes (*AtOPR1* and *AtOPR3)* from different subfamilies were proved to act in different biological processes or defense responses with tissue-specific expression patterns. The *AtOPR1* were mainly expressed in young rosettes, while the *AtOPR3* were predominantly expressed in young flowers [15,16]. The mutants of *AtOPR3* in subfamily II were male-sterile, and the OPDA (precursor of *AtOPR3*), when combined with ABA, could inhibit seed germination [16,26]. Besides, the *AtOPR3* was shown to function in the defense against nematodes through the JA and auxin signaling pathways in *Arabidopsis* [40]. These results demonstrated that the OPRs in subfamily II were essential for the male development and other physiological function. Furthermore, different expression patterns were identified in wheat *OPR* homoeologous genes, such as *TaOPRIII-13B* with *TaOPRIII-13A* and *TaOPRIII-13D*. These diverse gene expression patterns of *OPR* among homoeologous genes might be attributed to the loss or gain of gene function during the wheat evolution process [41]. In this study, the expression of *TaOPRII-1* was high in all the five tissues. Likewise, the *TaOPRIII-7* was highly expressed in root and leaf. Thus, it can be inferred that expression of wheat *OPR* genes exhibit a strongly tissue-specific pattern, which is in accordance with previous studies on *Arabidopsis*, rice, and maize [12,16,20].

The functions of OPRs in plant growth and development, as well as the defense responses to biotic and abiotic stresses, have been thoroughly investigated in *Arabidopsis* and tomato [18,26]. In this study, we proved that the expression of wheat OPRs could be induced to varied degrees by both biotic (aphid) and abiotic stress (drought, heat, salt, wounding) as well as exogenous hormone treatments (MeJA, ABA, and SA) (Figure 7). Furthermore, the TaOPRs could exhibit function diversity in response to various stresses. The expression of *TaOPRI-B2* (sub. I) and *TaOPRII-B1* (sub. II) were up-regulated when challenged with abiotic stresses (drought, heat, and wounding) and phytohormones (MeJA and ABA). However, the expression of *TaOPRIII-B7* (subfamily III), *TaOPRIV-A1* (subfamily IV), and *TaOPRV-B1* (subfamily V) decreased under abiotic (heat and salt) and biotic (aphid) stresses. The expressions of *TaOPRII-B1* in our trial (Figure 7) were consistent with previous results of *TaOPR2* (the same gene with *TaOPRII-B1*) under drought, salt, wounding, and MeJA treatments [22]. Exceptionally, Dong et al. [21] proved that the *TaOPR1* in subfamily I was up-regulated when challenged with salt stress, however in our study, the *TaOPRI-B2* (not the same gene as *TaOPR1*) was significantly down-regulated. As stated above, even if OPRs belonged to the same subfamily, they might exhibit inconsistent expression patterns under some stimulations. Beyond that, wheat varieties, as well as nutrient composition and contents, were varied between these investigations, and whether they influenced the responses remained inconclusive. More explorations were needed to illuminate the detailed mechanisms and other potential causes. Above all, the OPRs played multiple and vital roles in many biological processes of plants. For more example, the *OPR3* (OPRII) was essential for the embryo development of tomato [42]. Moreover, *OPR7* and *OPR8* (OPRII) could regulate the development and defensive responses of maize through the JA signaling pathway, and strong developmental defects were found in double mutant *opr7 opr8* (OPRII), such as the formation of a feminized tassel and the extreme elongation of ear shanks [19]. The OPRs in Sub. I and sub. II were found in both monocots and dicots, and they might exert basic and common functions, such as growth, development, and defense responses. However, the OPRs in subfamilies III, IV, and V only existed in monocots, and they were more likely to participate in specific signaling pathways and possess different functions.

The biological functions of OPRs in subfamilies III, IV, and V were less understood in comparison with those of subfamilies I and II. Rice *OPR1* (*OsOPR1*, sub. III) could participate in defensive responses to abiotic and biotic stresses (drought, salt, UV, heavy metals, fungus, O_3_ and, H_2_O_2_) as well as the stimulations of phytohormones (JA, ABA, SA, and ET) [43,44,45]. Besides, based on the analogous inducible transcription profiles, part of the rice OPRs of subfamily III and IV could exert similar functions as those of subfamily I and II, and they were considered as sub/neofunctionalized families [12]. To our knowledge, the *OPR* genes of subfamilies III, IV, and V in wheat have not been documented. The comprehensive analysis of the qRT-PCR results and public RNA-seq databases demonstrated that the *OPR* genes in sub. III, IV, and V could be induced by multiple stresses, and more investigations were needed to crosslink their multi-functions and detailed mechanisms.

## 4. Materials and Methods 

### 4.1. Identification of OPRs in Wheat

The protein sequences of dicot *Arabidopsis thaliana* (At), monocot *Oryza sativa* (Os), *Zea mays* (Zm), and *Triticum aestivum* (Ta) were obtained from public databases (*Arabidopsis thaliana* from TAIR, rice and maize from NCBI, and wheat from EnsemblPlants). At first, a local protein database was constructed using all the wheat protein sequences, which were downloaded from the Ensembl Plants of wheat (ftp://ftp.ensemblgenomes.org/pub/plants/release-42/fasta/triticum_aestivum). Then, the HMM profile of the family, *Oxidored_FMN* (PF00724), was downloaded from PFAM (http://pfam.xfam.org/), and this was used to search and compare the data with the local protein database, using the hmmsearch tool of the HMMER3.0 software. All the obtained protein sequences were verified by submitting them to the PFAM databases (http://pfam.xfam.org/) and NCBI Batch CD-search database (https://www.ncbi.nlm.nih.gov/Structure/bwrpsb/bwrpsb.cgi) to confirm the structural integrity of the *Oxidored*_FMN domain [46]. Finally, the physical and chemical properties of the putative TaOPR protein sequences, including the number of amino acids (NA), molecular weight (MW), and isoelectric point (theoretical pI), were calculated using the online ExPASy tool (https://web.expasy.org/protparam/) [47]. The subcellular localization of TaOPR proteins was predicted via the TargetP online server (http://www.cbs.dtu.dk/services/TargetP/).

### 4.2. Phylogenetic Analysis

Multiple alignments of the conserved TaOPR protein sequences were performed using ClustalW tool. The conserved protein blocks were selected using Gblocks Server (http://molevol.cmima.csic.es/castresana/Gblocks_server.html), which eliminated divergent regions and poorly aligned regions. The ProtTest v3.4 was used to select the best-fit model of amino acid substitution for tree-contracting analyses. The maximum-likelihood (ML) phylogenetic tree was constructed based on the conserved blocks and best-fit model using MEGA 7.0 software with 1000 bootstrap replications. 

### 4.3. Characterization of Gene Structure, Protein Domain, and Motif

The CDS and genomic sequences of *TaOPR* were acquired from Ensembl plants (http://plants.ensembl.org/Triticum_aestivum/Info/Index), and the exon-intron structures were displayed by searching the Gene Structure Display Server (GSDS) (http://gsds.cbi.pku.edu.cn/) [48]. The protein domains and motifs were discovered using an online tool, called the Multiple Expectation Maximization for Motif Elication (MEME Suite) (http://meme-suite.org/) [49].

### 4.4. Identification of Putative Cis-Acting Regulatory Elements

The 1.5 kb upstream sequences of all TaOPR transcripts were extracted, which were considered as the promoters for the prediction of *cis*-acting regulatory elements via PlantCARE (http://bioinformatics.psb.ugent.be/webtools/plantcare/html/). Virous *cis*-acting regulatory elements of all TaOPR transcripts were displayed in a diagram (Table S6).

### 4.5. Chromosomal Location of TaOPR Genes and Gene Duplication

To map the locations of putative *OPR* genes in the wheat chromosome, the initial chromosomal position and length of the chromosome of *OPR* genes were identified from the wheat genome database of Ensembl (http://plants.ensembl.org/Triticum_aestivum/Info/Index). Gene duplications of *TaOPR* were analyzed using the Multiple Collinearity Scan toolkit (MCScanx) (http://chibba.pgml.uga.edu/mcscan2/), and the E-value was 1 × 10^−10^. Tandem duplication of events were defined as two or more adjacent homologous genes located on one chromosome without any intervening gene, while segmentally duplicated genes were generated through polyploidy and experienced chromosome rearrangements [50]. The chromosomal distribution and segmental duplication of *TaOPR* genes was visualized by the CIRCOS program.

### 4.6. Expression Patterns of TaOPRs

To analyze the expression profiles of *TaOPR* genes in different stress challenges and tissues, the expression data were obtained from WheatExp (https://wheat.pw.usda.gov/WheatExp/) [51,52] and then visualized using the Heml software. The accession numbers and FPKM values of the *TaOPR* genes were presented in Tables S7 and S8.

### 4.7. Plant Materials and Treatments

The wheat cultivar “KN199” was grown in a controlled climate chamber (Boxun, BIC-300, Shanghai, China) under the following conditions: 25 °C/14 h light and 20 °C/10 h dark. The three-leaf-stage seedlings were used for tissue-specific analysis (leaf and root) and different stress treatments. Stress treatments included abiotic stress, hormones, and biotic stress. To induce abiotic stresses (salt, heat, drought, and wounding), seedlings were treated with a Hoagland liquid medium containing 200 mM NaCl for 1 h and 6 h (salt stress), 20% PEG6000 for 1 h and 6 h (drought stress), and 40 °C for 1 h and 6 h (heat stress) [53], respectively. For the wounding stress, leaves were cut with a blade and collected after 1h and 6 h. The seedlings were treated with 100 μM MeJA, 100 μM ABA, and 100 μM SA for 1 h and 6 h, respectively, and the leaves were then collected for further analysis of hormone challenges [21,22]. As for abiotic stress, 25 aphids (Greenbug, *Schizaphis graminum*) were placed on leaves for 12 h and 24 h of feeding [36]. Seedlings grown in a normal environment were used as a control. All the trials were performed with three biological replicates.

### 4.8. Determination of OPR mRNA Expression using qRT-PCR

The total RNA was extracted using the Takara RNA extraction Kit (Code No.9767, Japan), following the Instructions for Users. The first cDNA strand was synthesized using the Roche Transcriptor First Strand cDNA Synthesis Kit (Cat.No.04379012001, Germany). The qRT-PCR was performed using the CWBIO FastSYBR Mixture (No. CW0955, China) in a Roche LightCycler 480 System (Roche, Germany). A 20 μL reaction system was used, which can be described as follows: 10 μL 2×UltraSYBR Mixture (2×), 0.4 μL of forward primer (10.0 μmol/L), 0.4 μL of reverse primer (10.0 μmol/L), 1 μL (200 ng) of cDNA, and 8.2 μL of RNase-free water. The reaction procedure was completed in accordance with the following program: 95 °C for 10 min; 35 cycles of 95 °C for 15 s, 60 °C for 1 min; 95 °C for 15 s, 60 °C for 1 min, 95 °C for 15 s, and 60 °C for 15 s. Primer sequences for the *TaOPRI-B2*, *TaOPRII-B1*, *TaOPRIII-B7*, *TaOPRIV-A1*, *TaOPRV-B1*, and *TaActin*, used in this work, were presented in Table S9.

## 5. Conclusions

We identified and characterized the wheat *OPR* gene family for the first time in this study. A total of 48 TaOPRs were obtained and were classified into subfamilies I–V after systematic investigations. Tandem and segmental duplications contributed to the expansion of the *OPR* gene family, and segmental duplication tended to play the predominant role. The wheat OPRs were involved in crucial processes, such as plant growth and development, phytohormone-mediated metabolism, and defensive responses to various abiotic and biotic stresses. The expression patterns of *TaOPR* genes were tissue-specific and had stress-responsive diversity. This study could not only provide a scientific foundation for the comprehensive understanding of the wheat *OPR* gene family, but was also be helpful for screening more candidate genes and breeding new varieties of wheat with a high yield and stress resistance.

## Figures and Tables

**Table 1:** OPR-like genes in plants.

Lineage	Organism	Genome Size (Mbp)	Number	Nomenclature
Dicots	*Arabidopsis thaliana*	125	3	*AtOPR*
Monocots	*Oryza sativa*	430	13	*OsOPR*
	*Zea mays*	2365	8	*ZmOPR*
	*Triticum aestivum*	15800	48	*TaOPR*

## Supplementary Materials

Supplementary materials can be found at https://www.mdpi.com/1422-0067/20/8/1914/s1.
