# Supplementary material for: Genome-Wide Identification and Characterization of the OPR Gene Family in Wheat (Triticum aestivum L.)
Source: Int J Mol Sci. 2019 Apr 18;20(8):1914. doi: 10.3390/ijms20081914 (PMC6514991; doi:10.3390/ijms20081914)
Supplement: Supplementary file 1 [file ijms-20-01914-s001.zip › Additional File/Additional file 1:Table S1. Characteristics of the putative wheat OPR genes.pdf]

Additional File 1: Table S1 Characteristics of the putative wheat OPR genes.

No.	OPRs	Ensemble Wheat Gene ID	Subfamily	Subfamily ID	Amino acid length	PI	MW (kDa)	Subcellular location	Chromosome	Location
1	OPR1	TraesCS6B02G464800.1	OPRI	TaOPRI-B1	372	6.26	40.95	Chloroplast	6B	716009442-716015329
2	OPR2	TraesCS6B02G353200.1		TaOPRI-B2	336	6.76	37.24	Chloroplast	6B	618942386-618944489
3	OPR3	TraesCS6D02G302300.1		TaOPRI-D2	332	6.51	36.86	Mitochondrial	6D	411615155-411617388
4	OPR4	TraesCS2A02G311200.1		TaOPRI-A3	343	6.31	38.00	Mitochondrial	2A	534631870-534634396
5	OPR5	TraesCS2B02G328100.1		TaOPRI-B3	368	6.51	40.47	Chloroplast	2B	470065723-470070065
6	OPR6	TraesCS2D02G309400.1		TaOPRI-D3	370	6.21	40.88	Chloroplast	2D	396669711-396672291
7	OPR7	TraesCS7A02G412400.1	OPRII	TaOPRII-A1	390	8.23	42.89	Mitochondrial	7A	603291570-603295119
8	OPR8	TraesCS7B02G311600.1		TaOPRII-B1	390	8.23	42.78	Peroxisomal	7B	557057267-557060890
9	OPR9	TraesCS7D02G405500.1		TaOPRII-D1	390	8.23	42.86	Mitochondrial	7D	523045331-523049172
10	OPR10	TraesCS5B02G487600.1		TaOPRII-B2	381	5.77	41.72	Mitochondrial	5B	658542612-658548215
11	OPR11	TraesCS1A02G015700.1	OPRIII	TaOPRIII-A1	362	5.51	40.28	Mitochondrial	1A	8311562-8313243
12	OPR12	TraesCS1B02G018700.1		TaOPRIII-B1	362	5.57	40.18	Mitochondrial	1B	8913933-8916204
13	OPR13	TraesCS1D02G013600.1		TaOPRIII-D1	362	5.50	40.24	Mitochondrial	1D	6817464-6819186
14	OPR14	TraesCS1A02G015600.1		TaOPRIII-A2	397	5.49	44.05	Cytoplasmic	1A	8295508-8297343
15	OPR15	TraesCS1B02G019700.1		TaOPRIII-B2	399	5.58	44.14	Mitochondrial	1B	9263133-9265074
16	OPR16	TraesCS1D02G013500.1		TaOPRIII-D2	362	5.51	40.33	Mitochondrial	1D	6789468-6794893
17	OPR17	TraesCS1A02G015800.1		TaOPRIII-A3	356	5.69	39.64	Cytoplasmic	1A	8320695-8322586
18	OPR18	TraesCS1B02G019800.1		TaOPRIII-B3	362	5.79	40.27	Mitochondrial	1B	6864265-6866168
19	OPR19	TraesCS1D02G013700.1		TaOPRIII-D3	362	5.59	40.25	Mitochondrial	1D	6862265-6866168
20	OPR20	TraesCS4B02G048500.1		TaOPRIII-B4	362	5.79	40.32	Mitochondrial	4B	36762020-36763524
21	OPR21	TraesCS1D02G013000.1		TaOPRIII-D5	362	5.90	40.29	Mitochondrial	1D	6731413-6732961
22	OPR22	TraesCS1A02G217700.1		TaOPRIII-A6	361	5.74	40.25	Cytoplasmic	1A	385660663-385662337
23	OPR23	TraesCS1B02G231000.1		TaOPRIII-B6	279	5.98	30.76	Cytoplasmic	1B	414806177-414807939
24	OPR24	TraesCS1D02G219500.1		TaOPRIII-D6	366	5.75	40.73	Cytoplasmic	1D	307217310-307219380
25	OPR25	TraesCS2A02G026600.1		TaOPRIII-A7	369	6.01	40.84	Cytoplasmic	2A	12287484-12289174
26	OPR26	TraesCS2B02G040000.1		TaOPRIII-B7	369	6.01	40.71	Cytoplasmic	2B	18177103-18178722
27	OPR27	TraesCS2D02G028600.1		TaOPRIII-D7	330	6.39	36.60	Cytoplasmic	2D	11774695-11775912
28	OPR28	TraesCS1A02G015500.1		TaOPRIII-A8	379	6.14	41.00	Cytoplasmic	1A	8291349-8292883
29	OPR29	TraesCS1B02G019600.1		TaOPRIII-B8	332	7.73	36.58	Mitochondrial	1B	9260224-9261405
30	OPR30	TraesCS1D02G013400.1		TaOPRIII-D8	382	5.81	41.40	Cytoplasmic	1D	6786398-6787884
31	OPR31	TraesCS1B02G018800.1		TaOPRIII-B9	314	5.81	34.49	Peroxisomal	1B	8917761-8918856
32	OPR32	TraesCS7B02G455400.1		TaOPRIII-B10	394	5.85	42.97	Cytoplasmic	7B	716132585-716134045
33	OPR33	TraesCS7D02G524600.1		TaOPRIII-D10	342	5.55	37.27	Cytoplasmic	7D	620475377-620476896
34	OPR34	TraesCS7A02G537100.1		TaOPRIII-A11	342	5.45	37.25	Mitochondrial	7A	714616516-714618249
35	OPR35	TraesCS7B02G455300.1		TaOPRIII-B11	343	5.38	37.32	Mitochondrial	7B	716041058-716042585
36	OPR36	TraesCS7D02G524400.1		TaOPRIII-D11	343	5.45	37.46	Cytoplasmic	7D	620281144-620282880
37	OPR37	TraesCS7A02G174500.1		TaOPRIII-A12	334	5.57	37.40	Cytoplasmic	7A	128404791-128406105
38	OPR38	TraesCS7B02G079300.1		TaOPRIII-B12	380	8.16	43.13	Cytoplasmic	7B	89541640-89543039
39	OPR39	TraesCS7D02G175600.1		TaOPRIII-D12	204	5.48	23.64	Mitochondrial	7D	127649536-127651843
40	OPR40	TraesCS7A02G174700.1		TaOPRIII-A13	369	5.65	41.18	Mitochondrial	7A	128509817-128511499
41	OPR41	TraesCS7B02G079500.1		TaOPRIII-B13	368	5.62	41.19	Mitochondrial	7B	89655918-89657599
42	OPR42	TraesCS7D02G175900.1		TaOPRIII-D13	275	5.09	30.54	Peroxisomal	7D	127752063-127753820
43	OPR43	TraesCS7D02G175800.1		TaOPRIII-D14	299	5.43	33.09	Mitochondrial	7D	127699170-127700372
44	OPR44	TraesCS5A02G525300.1	OPRIV	TaOPRIV-A1	365	5.58	40.24	Mitochondrial	5A	685790993-685794738
45	OPR45	TraesCS4B02G356100.1		TaOPRIV-B2	391	5.80	43.30	Cytoplasmic	4B	646862886-646864601
46	OPR46	TraesCS4D02G349500.1		TaOPRIV-D2	366	5.50	40.60	Cytoplasmic	4D	502488484-502490376
47	OPR47	TraesCS2B02G328200.1	OPRV	TaOPRV-B1	370	4.99	41.14	Cytoplasmic	2B	470070583-470072507
48	OPR48	TraesCS2D02G309500.1		TaOPRV-D1	370	4.84	41.10	Cytoplasmic	2D	396712074-396713976
