# Supplementary material for: Genome-Wide Identification and Characterization of the OPR Gene Family in Wheat (Triticum aestivum L.)
Source: Int J Mol Sci. 2019 Apr 18;20(8):1914. doi: 10.3390/ijms20081914 (PMC6514991; doi:10.3390/ijms20081914)
Supplement: Supplementary file 1 [file ijms-20-01914-s001.zip › Additional File/Additional File 2:Table S2 The gene ID of Arabidopsis, maize and rice.pdf]

Additional File 2: Table S2 The OPR gene IDs of *Arabidopsis* , maize and rice.

No.	OPRs	Gene ID	OPRs	Gene ID	OPRs	Gene ID
1	AtOPR1	AT1G76680	ZmOPR1	AAY26521.1	OsOPR1	Q84QK0.1
2	AtOPR2	AT1G76690	ZmOPR2	AAY26522.1	OsOPR2	Q69TH4.1
3	AtOPR3	AT2G06050	ZmOPR3	AAY26523.1	OsOPR3	Q69TH6.1
4			ZmOPR4	AAY26524.1	OsOPR4	Q69TH8.1
5			ZmOPR5	AAY26525.1	OsOPR5	Q69TI0.1
6			ZmOPR6	AAY26526.1	OsOPR6	Q69TI2.1
7			ZmOPR7	AAY26527.1	OsOPR7	Q6Z965.1
8			ZmOPR8	AAY26528.1	OsOPR8	Q0E0C6.1
9					OsOPR9	Q5ZC83.1
10					OsOPR10	Q0JMR0.1
11					OsOPR11	B9FSC8.1
12					OsOPR12	B9FFD2.2
13					OsOPR13	B9FFD3.1
