# Supplementary material for: Genome-Wide Identification and Characterization of the OPR Gene Family in Wheat (Triticum aestivum L.)
Source: Int J Mol Sci. 2019 Apr 18;20(8):1914. doi: 10.3390/ijms20081914 (PMC6514991; doi:10.3390/ijms20081914)
Supplement: Supplementary file 1 [file ijms-20-01914-s001.zip › Additional File/Additional File 3:Table S3 Detail information of the conserved motifs of TaOPRs.pdf]

Additional File 3: Table S3 Detail information of the conserved motifs of TaOPRs.

Logo	Name	E-value	Alt. Name	Width
1	MEME-1	3.9e-1787	RNAIEAGFDGVEIHGAHGYJIEQFLKDSANDRTDEY GGSLENRCRFALEV	50
2	MEME-2	9.3e-1493	YPDTPGIWTAEQVEAWKPIVDAVHAKGALFFCQJ WHVGRVS	41
3	MEME-3	2.2e-1467	GYADLVAYGRLFLANPDLPKRFELGAPLNKYDRM TFYTSDP	41
4	MEME-4	2.1e-1580	FDLAHRVVLAPLTRQRSYGNVPQPHAALYYSQRA TKGGLLIAEATGVSDT	50
5	MEME-5	3.4e-1121	AVVKEVGAHRVGIRLSPFADYMDCHDSDPHALAL YMATKLND	42
6	MEME-6	1.5e-875	VPHRLLPYREAFKGTFIANGGYDREEGNK	29
7	MEME-7	9.9e-695	DGRLEEFSPPRRLATEEIPAIVDDFRKAA	29
8	MEME-8	9.9e-419	TYEFQPGGAAPJSSTDKGVGP	21
9	MEME-9	1.2e-411	HGILYCHMIEPRMAI	15
10	MEME-10	1.2e-349	VVGYTDYPFLE	11
