# Supplementary material for: Genome-Wide Identification and Characterization of the OPR Gene Family in Wheat (Triticum aestivum L.)
Source: Int J Mol Sci. 2019 Apr 18;20(8):1914. doi: 10.3390/ijms20081914 (PMC6514991; doi:10.3390/ijms20081914)
Supplement: Supplementary file 1 [file ijms-20-01914-s001.zip › Additional File/Additional File 4:Table S4 Tandem duplicated TaOPR gene pairs..pdf]

Additional File 4: Table S4 Tandem duplicated TaOPR gene pairs.

Gene ID	Gene Name	Gene ID	Gene Name
TraesCS1A02G015500	TaOPRIII-A8	TraesCS1A02G015600	TaOPRIII-A2
TraesCS1A02G015600	TaOPRIII-A2	TraesCS1A02G015700	TaOPRIII-A1
TraesCS1B02G019600	TaOPRIII-B8	TraesCS1B02G019700	TaOPRIII-B2
TraesCS1B02G019700	TaOPRIII-B2	TraesCS1B02G019800	TaOPRIII-B3
TraesCS1D02G013500	TaOPRIII-D2	TraesCS1D02G013600	TaOPRIII-D1
TraesCS7A02G174500	TaOPRIII-A12	TraesCS7A02G174700	TaOPRIII-A13
TraesCS7B02G455300	TaOPRIII-B11	TraesCS7B02G455400	TaOPRIII-B10
TraesCS7D02G524400	TaOPRIII-D11	TraesCS7D02G524600	TaOPRIII-D10
