# Supplementary material for: Genome-Wide Identification and Characterization of the OPR Gene Family in Wheat (Triticum aestivum L.)
Source: Int J Mol Sci. 2019 Apr 18;20(8):1914. doi: 10.3390/ijms20081914 (PMC6514991; doi:10.3390/ijms20081914)
Supplement: Supplementary file 1 [file ijms-20-01914-s001.zip › Additional File/Additional File 5:Table S5 Segmentally duplicated TaOPR gene pairs..pdf]

Additional File 5: Table S5 Segmentally duplicated TaOPR gene pairs.

No.	Gene ID	Gene Name	Gene Position	Gene ID	Gene Name	Gene Position
1	TraesCS1A02G015500	TaOPRIII-A8	1A	TraesCS1B02G019600	TaOPRIII-B8	1B
2	TraesCS1A02G015600	TaOPRIII-A2	1A	TraesCS1B02G019700	TaOPRIII-B2	1B
3	TraesCS1A02G015600	TaOPRIII-A2	1A	TraesCS1D02G013500	TaOPRIII-D2	1D
4	TraesCS1A02G015700	TaOPRIII-A1	1A	TraesCS1D02G013000	TaOPRIII-D5	1D
5	TraesCS1A02G015800	TaOPRIII-A3	1A	TraesCS1B02G019800	TaOPRIII-B3	1B
6	TraesCS1A02G015800	TaOPRIII-A3	1A	TraesCS1D02G013700	TaOPRIII-D3	1D
7	TraesCS1A02G217700	TaOPRIII-A6	1A	TraesCS1B02G231000	TaOPRIII-B6	1B
8	TraesCS1A02G217700	TaOPRIII-A6	1A	TraesCS1D02G219500	TaOPRIII-D6	1D
9	TraesCS1B02G018700	TaOPRIII-B1	1B	TraesCS1D02G013000	TaOPRIII-D5	1D
10	TraesCS1B02G019600	TaOPRIII-B8	1B	TraesCS1D02G013400	TaOPRIII-D8	1D
11	TraesCS1B02G019800	TaOPRIII-B3	1B	TraesCS1D02G013500	TaOPRIII-D2	1D
12	TraesCS1B02G231000	TaOPRIII-B6	1B	TraesCS1D02G219500	TaOPRIII-D6	1D
13	TraesCS1A02G015500	TaOPRIII-A8	1A	TraesCS1D02G013400	TaOPRIII-D8	1D
14	TraesCS2A02G026600	TaOPRIII-A7	2A	TraesCS2B02G040000	TaOPRIII-B7	2B
15	TraesCS2A02G026600	TaOPRIII-A7	2A	TraesCS2D02G028600	TaOPRIII-D7	2D
16	TraesCS2A02G311200	TaOPRI-A3	2A	TraesCS2B02G328100	TaOPRI-B3	2B
17	TraesCS2A02G311200	TaOPRI-A3	2A	TraesCS2D02G309400	TaOPRI-D3	2D
18	TraesCS2B02G040000	TaOPRIII-B7	2B	TraesCS2D02G028600	TaOPRIII-D7	2D
19	TraesCS2B02G328100	TaOPRI-B3	2B	TraesCS2D02G309400	TaOPRI-D3	2D
20	TraesCS2B02G328200	TaOPRV-B1	2B	TraesCS2D02G309500	TaOPRV-D1	2D
21	TraesCS4B02G356100	TaOPRIV-B2	4B	TraesCS4D02G349500	TaOPRIV-D2	4D
22	TraesCS4B02G356100	TaOPRIV-B2	4B	TraesCS5A02G525300	TaOPRIV-A1	5A
23	TraesCS4D02G349500	TaOPRIV-D2	4D	TraesCS5A02G525300	TaOPRIV-A1	5A
24	TraesCS6B02G353200	TaOPRI-B2	6B	TraesCS6D02G302300	TaOPRI-D2	6D
25	TraesCS7A02G174500	TaOPRIII-A12	7A	TraesCS7B02G079300	TaOPRIII-B12	7B
26	TraesCS7A02G174500	TaOPRIII-A12	7A	TraesCS7D02G175600	TaOPRIII-D12	7D
27	TraesCS7A02G412400	TaOPRII-A1	7A	TraesCS7B02G311600	TaOPRII-B1	7B
28	TraesCS7A02G412400	TaOPRII-A1	7A	TraesCS7D02G405500	TaOPRII-D1	7D
29	TraesCS7A02G537100	TaOPRIII-A11	7A	TraesCS7B02G455300	TaOPRIII-B11	7B
30	TraesCS7A02G537100	TaOPRIII-A11	7A	TraesCS7D02G524400	TaOPRIII-D11	7D
31	TraesCS7B02G079300	TaOPRIII-B12	7B	TraesCS7D02G175600	TaOPRIII-D12	7D
32	TraesCS7B02G311600	TaOPRII-B1	7B	TraesCS7D02G405500	TaOPRII-D1	7D
