# Supplementary material for: Genome-Wide Identification and Characterization of the OPR Gene Family in Wheat (Triticum aestivum L.)
Source: Int J Mol Sci. 2019 Apr 18;20(8):1914. doi: 10.3390/ijms20081914 (PMC6514991; doi:10.3390/ijms20081914)
Supplement: Supplementary file 1 [file ijms-20-01914-s001.zip › Additional File/Additional File 7:Table S7 FPKM values of 46 TaOPR genes in five tissues (leaf, root, spike, stem and grain) in this study..pdf]

Additional File 7: Table S7 FPKM values of 46 TaOPR genes in five tissues (leaf, root, spike, stem and grain) in this study.

Gene	leaf_z10	root_z10	spike_z32	stem_z32	grain_z71
TaOPRI-B1	0.06	0.05	104.8	137.08	0.31
TaOPRI-B2	0.68	3.36	2.25	0	4.26
TaOPRI-D2	0.85	1.4	3.51	0.07	7.35
TaOPRI-A3	6.44	13.83	27.69	10.9	15.53
TaOPRI-B3	1.16	0.93	3.54	2.12	2.47
TaOPRI-D3	1.82	2.61	4.71	4.57	3.16
TaOPRII-A1	14.97	19.64	25.87	11.76	24.68
TaOPRII-B1	12.63	16.71	19.89	8.56	26.31
TaOPRII-D1	14.97	19.64	25.87	11.76	24.68
TaOPRII-B2	0	0	0.19	0	0
TaOPRIII-D14	2.06	31.9	0.85	0.29	15.25
TaOPRIII-A13	1.02	7.03	0.24	0.03	2.05
TaOPRIII-B13	2.06	31.9	0.85	0.29	15.25
TaOPRIII-D13	0.51	3.07	0.1	0	1.51
TaOPRIII-A12	0.02	0.33	0.32	1.76	0.03
TaOPRIII-B12	0.17	1.89	1.66	3.82	0.12
TaOPRIII-D12	0.02	0.33	0.32	1.76	0.03
TaOPRIII-D11	1.06	2.57	3.35	2.24	2.45
TaOPRIII-B10	1.06	2.57	3.35	2.24	2.45
TaOPRIII-D10	1.06	2.57	3.35	2.24	2.45
TaOPRIII-B9	0	0.1	0	0	0.05
TaOPRIII-A8	0	0.44	0.02	0	0.01
TaOPRIII-B8	0.02	0.06	0.07	0.04	0.11
TaOPRIII-D8	0	0.1	0	0	0.05
TaOPRIII-A7	5.6	21.95	0.51	0.17	0.74
TaOPRIII-B7	11.53	53.59	1.26	0.84	0.56
TaOPRIII-D7	11.53	53.59	1.26	0.84	0.56
TaOPRIII-A6	1.08	0.52	0.37	0.09	0.5
TaOPRIII-B6	0.02	0.11	0.04	0.13	0.1
TaOPRIII-D6	1.08	0.52	0.37	0.09	0.5
TaOPRIII-D5	0.1	29.87	0.24	0.04	0.04
TaOPRIII-B4	0.12	20.81	0.51	0	0.02
TaOPRIII-A3	0.01	0.47	0.05	0.38	0.01
TaOPRIII-B3	0.07	0.25	0.34	0.18	0.05
TaOPRIII-D3	0.04	0.06	0.37	0.13	0.09

TaOPRIII-A2	0.04	4.34	0.67	0	0
TaOPRIII-B2	0.03	2.42	0.17	0.12	0.07
TaOPRIII-D2	0	16.03	0	0	0
TaOPRIII-A1	0.02	2.03	0.02	0	0
TaOPRIII-B1	0.03	2.42	0.17	0.12	0.07
TaOPRIII-D1	0	2.63	0.16	0	0
TaOPRIV-A1	0	0.2	1.48	8.35	0.72
TaOPRIV-B2	0	0.2	1.48	8.35	0.72
TaOPRIV-D2	0	0.2	1.48	8.35	0.72
TaOPRV-B1	0.68	0.06	0.79	0.15	9.71
TaOPRV-D1	0.68	0.06	0.79	0.15	9.71
TaOPRV-2	0.68	0.06	0.79	0.15	9.71
