# Supplementary material for: Genome-Wide Identification and Characterization of the OPR Gene Family in Wheat (Triticum aestivum L.)
Source: Int J Mol Sci. 2019 Apr 18;20(8):1914. doi: 10.3390/ijms20081914 (PMC6514991; doi:10.3390/ijms20081914)
Supplement: Supplementary file 1 [file ijms-20-01914-s001.zip › Additional File/Additional File 8:Table S8 FPKM values of 46 TaOPR genes under two abiotic stresses (drought and heat) in this study..pdf]

Additional File 8: Table S8 FPKM values of 46 TaOPR genes under two abiotic stresses (drought and heat) in this study.

Gene	Drought_1hr	Drought_6hr	Control	Heat1_hr	Heat_6hr
TaOPRI-B1	0.04	0.03	0.06	0.03	0.02
TaOPRI-B2	71.47	34.1	87.07	62.32	79.51
TaOPRI-D2	27.52	19.24	33.24	51.48	69.11
TaOPRI-A3	44.71	34.96	55.89	37.78	138.65
TaOPRI-B3	7.07	4.39	6.43	3.98	9.78
TaOPRI-D3	9.76	10.11	9.84	4.9	11.6
TaOPRII-A1	17.37	21.3	15.19	22.13	57.93
TaOPRII-B1	14.81	21.74	14.33	18.41	47.2
TaOPRII-D1	17.37	21.3	15.19	22.13	57.93
TaOPRII-B2	0.02	0	0.02	0	0
TaOPRIII-D14	9.51	3.21	3.16	8.61	3.93
TaOPRIII-A13	1.25	0.3	0.54	9.87	0.83
TaOPRIII-B13	9.51	3.21	3.16	8.61	3.93
TaOPRIII-D13	5.01	0.38	1	93.02	5.4
TaOPRIII-A12	0.06	0.07	0.13	0.02	0.19
TaOPRIII-B12	0.49	0.16	0.71	0.02	0.31
TaOPRIII-D12	0.06	0.07	0.13	0.02	0.19
TaOPRIII-D11	11.04	45.5	7.81	6.13	6.83
TaOPRIII-B10	0.82	0.32	2.45	1.06	0.84
TaOPRIII-D10	11.04	45.5	7.81	6.13	6.83
TaOPRIII-B9	0.12	0.18	0.26	0.03	0.02
TaOPRIII-A8	0.07	0.15	0.19	0.11	0.01
TaOPRIII-B8	0	0.04	0.06	0.27	0
TaOPRIII-D8	0.12	0.18	0.26	0.03	0.02
TaOPRIII-A7	16.2	0.85	21.49	13.01	2.6
TaOPRIII-B7	39.27	6.3	43.98	30.24	7.04
TaOPRIII-D7	39.27	6.3	43.98	30.24	7.04
TaOPRIII-A6	0.3	0.21	0.41	0.11	0.33
TaOPRIII-B6	0.52	1.42	0.27	0.1	0.17
TaOPRIII-D6	0.3	0.21	0.41	0.11	0.33
TaOPRIII-D5	0.04	0.1	0.02	0.02	0.05
TaOPRIII-B4	0.03	0.02	0	0	0.02
TaOPRIII-A3	0.04	0	0.04	0.02	0
TaOPRIII-B3	10.23	1.24	10.63	6.84	1.32
TaOPRIII-D3	2.79	0.85	1.81	1.48	0.11

TaOPRIII-A1	0.1	0.04	0.03	0.25	0.03
TaOPRIII-B1	0.04	0.23	0.2	0.1	0.05
TaOPRIII-D1	0.39	0.15	0.74	0.86	0.25
TaOPRIII-A2	0.01	0	0.02	0	0
TaOPRIII-B2	0.04	0.23	0.2	0.1	0.05
TaOPRIII-D2	0.03	0	0	0.07	0
TaOPRIV-A1	0.51	2.35	0.64	0.19	0.52
TaOPRIV-B2	0.51	2.35	0.64	0.19	0.52
TaOPRIV-D2	0.51	2.35	0.64	0.19	0.52
TaOPRV-B1	0.2	0.1	0.09	0.11	0.04
TaOPRV-D1	0.2	0.1	0.09	0.11	0.04
TaOPRV-2	0.2	0.1	0.09	0.11	0.04
