# Supplementary material for: Genome-Wide Identification and Characterization of the OPR Gene Family in Wheat (Triticum aestivum L.)
Source: Int J Mol Sci. 2019 Apr 18;20(8):1914. doi: 10.3390/ijms20081914 (PMC6514991; doi:10.3390/ijms20081914)
Supplement: Supplementary file 1 [file ijms-20-01914-s001.zip › Additional File/Additional File 9:Table S9 The sequences of the qRT-PCR primers in this study.pdf]

Additional File 9: Table S9 The sequences of the qRT-PCR primers in this study.

Primer name	Primer sequence
OPRI-B2F	TCAAGGACGGCATAAACGACC
OPRI-B2R	CGAGTCGTAAGCGTCAAGGTG
OPRII-B1F	TTCTCGCCGTACCAGATGC
OPRII-B1R	GGAGCGCTGCGAGTAGTACTC
OPRIII-B7F	ATCGTCGACGACTTCAGGAA
OPRIII-B7R	GCGCTGTCCTTGAGGAACT
OPRIV-A1F	AGGCTACGCTGATCTTGTGG
OPRIV-A1R	GGGTAGTCGGTGTAGCCAAC
OPRV-B1F	CAAGAACCCATCTCCAGCA
OPRV-B1R	CGGCTTCAAGAGCATTTCGG
TaActin-F	TAGATGCAGTAAAGAACCTGAC
TaActin-R	GCCGTGGAGAAGAAGGATC
